# Development and Characterization of Liposomal Doxorubicin Hydrochloride with Palm Oil

**DOI:** 10.1155/2014/765426

**Published:** 2014-03-27

**Authors:** Bahareh Sabeti, Mohamed Ibrahim Noordin, Shaharuddin Mohd, Rosnani Hashim, Afendi Dahlan, Hamid Akbari Javar

**Affiliations:** ^1^Department of Pharmacy, Faculty of Medicine, University of Malaya, 51200 Kuala Lumpur, Malaysia; ^2^Faculty of Pharmacy, Cyberjaya University College of Medical Sciences, 63000 Cyberjaya, Selangor, Malaysia; ^3^Department of Pharmaceutics, Faculty of Pharmacy, Tehran University of Medical Sciences, Tehran 141176, Iran

## Abstract

The usage of natural products in pharmaceuticals has steadily seen improvements over the last decade, and this study focuses on the utilization of palm oil in formulating liposomal doxorubicin (Dox). The liposomal form of Dox generally minimizes toxicity and enhances target delivery actions. Taking into account the antiproliferative and antioxidant properties of palm oil, the aim of this study is to design and characterize a new liposomal Dox by replacing phosphatidylcholine with 5% and 10% palm oil content. Liposomes were formed using the freeze_thaw method, and Dox was loaded through pH gradient technique and characterized through *in vitro* and *ex vivo* terms. Based on TEM images, large lamellar vesicles (LUV) were formed, with sizes of 438 and 453 nm, having polydispersity index of 0.21 ± 0.8 and 0.22 ± 1.3 and zeta potentials of about −31 and −32 mV, respectively. In both formulations, the entrapment efficiency was about 99%, and whole Dox was released through 96 hours in PBS (pH = 7.4) at 37°C. Comparing cytotoxicity and cellular uptake of LUV with Caelyx^R^ on MCF7 and MDA-MBA 231 breast cancer cell lines indicated suitable uptake and lower IC50 of the prepared liposomes.

## 1. Introduction

Doxorubicin hydrochloride (Dox) is an antitumor antibiotic derived from anthracyclines. While the usage of anthracyclines is limited due to their dose-related cardiotoxicity and myelosuppression, applying liposomal Dox in ovary, lung, and breast cancer therapies has been approved by WHO due to its superior efficacy and minimum cardiotoxicity [1, 2]. Furthermore, the liposomal forms allow Dox to remain in the circulation system for longer periods of time, which will allow for the delivery of a greater amount of the drug to cancerous cells or tumors [1, 3, 4]. Both the prolonged exposure of tumor cells to liposome and the capability to distinguish the differential between tumors via tissue cells are valuable reasons to develop liposomes. On the other hand, as nanoparticles are regarded as a valuable carrier, nanoliposome is also one of the well-known and established developments in drug delivery systems [5].

Since palm oil has antiproliferative and antioxidant properties due to presence of components such as carotenes, tocopherol, tocotrienols, terpenoids, and flavonoids, it is viable for use in pharmaceutical products, on top of its nutritional advantages. In addition, its antioxidants help resist rancidity and improve the stability of palm oil [6–8].

Considering the anticancer properties of palm oil and great advantages of liposome, the aim of this study was to prepare liposomal Dox by applying palm oil fractions.

## 2. Materials

Doxorubicin hydrochloride (Dox), palm oil (Po), cholesterol (CH), L-alpha-phosphatidylcholine (PC), polyethylene glycol (PEG), methanol, and chloroform were purchased from Sigma-Aldrich (Germany). Sodium hydroxide and potassium dihydrogen phosphate were purchased from Merck (Germany).

## 3. Method

Liposomes were prepared using the freeze-thaw method and pH gradient technique, carried out in order to maximize the loading of Dox within liposome [9–11]. Two formulations were designed; both consisted of 45 mg CH and 5 mg PEG with different percentages of PC and palm oil. The first formula (Fa) contains 5% palm oil and 45% of PC, while the second formula (Fb) contains 10% palm oil and 40% PC in their respective formulations. Then, all of the lipid components and PEG were dissolved in a chloroform : methanol mixture of (2 : 1, v/v) in a round-bottom flask. The solvent was removed under vacuum using a rotary evaporator (Rotavapour R-124, BÜCHI) at 40°C and 50 rpm. After a thin lipid film was formed in the interior of the flask, the system was purged with nitrogen to remove organic solvent entirely. The lipid film layer was hydrated with 10 mL Citrate buffered solution (pH = 4) and then sonicated for 30 minutes in a bath type sonicator (Sonicor). The freeze-thaw cycle was carried out five times via freezing under −80°C and then heated mixture in water bath at 65°C with the intention of decreasing the size, further entrapping the acidic buffer inside the liposome. Bicarbonate buffer (pH = 0.5) was added dropwise to the mixture (for the reason of adjusting outer liposomes space into a physiological pH) until its pH reaches 7. Afterwards, 10 mL of Dox medium in distilled water (2000 *μ*g/mL) was added to the mixture and shaken at room temperature for 30 minutes at 60 rmp [12–14].

### 3.1. Formation and Morphology

The formation of liposomes was observed with a transmission electron microscope (TEM). Samples were prepared by applying a drop of the mixture to a carbon-coated copper grid and left for a minute to allow some of the particles to adhere onto the carbon substrate. After removing the excess dispersion with a piece of filter paper, a drop of 1% phosphotungstic acid solution was applied for one minute and then left to be air-dried. The samples were viewed with a TEM (ABFETEM Leo 9112) [14–17].

### 3.2. Particle Size Distribution, Polydispersity Index (PDI), and Zeta Potential (ZP) Measurement

To evaluate the size distribution, PDI, and value ZP of each sample, 50 mg of liposome was weighted and dispersed in 20 mL distilled water and then those parameters were measured by the zetasizer (Zetasizer Nanoseries, Malbern Instrument) [18–20]. This test was repeated thrice.

### 3.3. Construction of Standard Curve

Dilutions of doxorubicin HCL were in the range of 400, 200, 100, 50, 25, and 12.5 ng/mL, prepared and detected by HPLC with a fluorescence detector (Chromolith Performance RP-8e 100 mm × 4.6 mm column protected by a Chromolith Guard Cartridge RP-18e 5 mm × 4.6 mm, Merck, Darmstadt, Germany).

*Mixture of Acetonitrile*. Heptanesulfonic acid (0.2%, pH 4) by a ratio of 25 : 75 was applied as mobile phase with the flow rate of 1 mL/min.

Dox has an excitation wavelength (*λ*_ex_) of 480 nm and an emission wavelength (*λ*_em_) of 560 nm. The calibration curve was constructed using the Microsoft Excel 2007 program [19, 21].

### 3.4. Evaluation of Entrapment Efficiency and* In Vitro* Release

The mixture was centrifuged (Universal 32) for 70 minutes at 14000 rpm, the supernatant containing free Dox was obtained, and the absorbance was measured using HPLC [15]. The entrapment efficiency of liposomes was determined by the following formula:

(1) $$\begin{array}{c} \text{EE}\left(\%\right)=\left\{\frac{\left(C_{i}-C_{f}\right)}{C_{i}}\right\}\times 100, \end{array}$$

where EE is the concentration of entrapped drug (ng/mL), *C*_*i*_ is the initial concentration of drug used in formulating the liposomes (ng/mL), *C*_*f*_ is the concentration of drug in the supernatant (ng/mL), and EE (%) is the percentage of the drug's entrapment.

To estimate the* in vitro* drug release of liposomal Dox, a dialysis bag was used [22]. After separating free drug, 100 mg of liposome was weighted and then placed directly into the dialysis bag (Mw12000). The dialysis bag was sealed at both ends and located in a 1000 mL fresh PBS buffer medium (pH 7.4) at 37°C, at 90 rpm under perfect sink conditions [15, 23, 24]. At predetermined time intervals, 1 mL of the medium was sampled for further analysis by HPLC. The concentrations of doxorubicin throughout the releasing time were calibrated using the calibration equation. The results recorded are the mean value of the three runs carried out for each liposome concentration. The percentage of released Dox at certain time was plotted using Microsoft Excel 2007 and was defined by the following formula:

(2) $$\begin{array}{c} \text{Drug}\;\text{release}\left(\%\right)=\left(\frac{C_{t}\times 10^{-3}}{C_{i}}\right)\times 100, \end{array}$$

where *C*_*t*_ is the concentration of drug released (ng/mL) at time *t* and *C*_*i*_ is the initial drug concentration (ng/mL).

### 3.5. Cellular Uptake

To observe the cellular uptake, two breast cancer cell lines, received from Pasture Institute, were utilized separately. MCF-7 cells were cultured in RPMI 1640, and 10% FBS were then seeded in 24-well plates with a density of 1 × 10^5^ cells/well and incubated in 37°C with 5% CO_2 _for 24 h. 50 *μ*L Dox liposome (2000 *μ*g/mL) was added into each well and incubated for 24 h, and then the cells were washed thrice with BPS, respectively. Afterward, image analyses of cells were performed with confocal microscopy (IX71, Olympus, Japan), and the same procedure was carried out for MDA-MBA, 231 cells as well [25, 26].

### 3.6. Cytotoxicity Assay

MTT assay was performed to observe the cytotoxic activities of designed liposome, IC50 of formulation assessed in cell culture media and compared with Caelyx^R^ (pegylated liposomal Dox). The human breast cell lines MCF 7 and MDA-MBA 231 were seeded in 96-well plates with a density of 7 × 10^3^ cells/well, using RPMI 1640 and 10% FBS added and then incubated in 37°C with 5% CO_2 _for 24 h. The cells were then treated with various concentrations of Caelyx^R^ (2000 *μ*g/mL), Fa, and Fb (liposome containing 5% and 10% of palm oil loaded with 2000 *μ*g/mL doxorubicin, which is in the same concentration of Caelyx^R^), respectively, and then incubated for 48 h. Afterwards, the media were removed, and 10 *μ*L MTT was added to each well, incubated for a further four hours. Finally, the MTT was removed, and 100 *μ*L DMSO was added to each well, and the absorbance was measured with an ELISA reader at *λ* = 595 nm [27–29].

### 3.7. Statistical Analysis

All of the results were remarked with the mean ± SD, and the one-way analysis of variance (ANOVA) was employed for statistical analysis of the data, followed by Scheffe post hoc test using (SPSS 15 for Windows 7). Differences were considered significant at *P* < 0.05.

## 4. Results and Discussion

### 4.1. Liposomes Formation and Morphology

**Figure 1:**
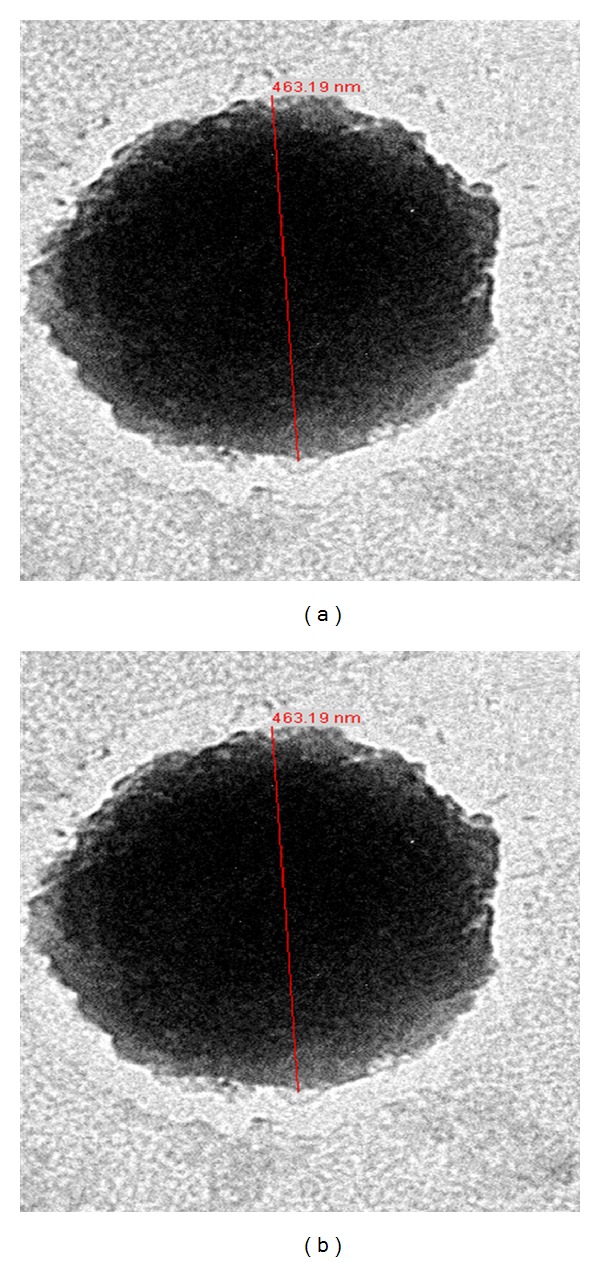
TEM images of Dox liposome with magnification 8000x, (a) (Fa), (b) (Fb).

TEM images in Figure 1 demonstrate the formation of vesicles. Considering the TEM images, one layer liposome with large inside capacity confirms the fine formation and well shape of the LUV in both formulations.

### 4.2. Particle Size Distribution and Zeta Potential Measurement

Particle size determinations are mostly performed to confirm that the desired liposome size range has been obtained during preparation because suitable size of particles is important for their interaction with the biological situation; for instance, through intravenous administration of loaded particles, their ability to pass or leave the vascular capillaries effectively is dependent on the sizing [18, 30]. Referring to Table 1, Fa has a size of 438.74 ± 1.9 nm, while Fb has a size of 453.71 ± 1.1 nm; the nanosize of LUVs would result in advance drug delivery.

The polydispersity index value is a measure of the heterogeneity of particle sizes in a compound. Liposomes with PDI value between 0.1 and 0.25 display more uniformity and physical stability. Further PDI value more than 0.5 indicates the poor uniformity of mixture [20, 31]. Looking at Table 1, the PDI values of liposomes are 0.22 ± 1.3 and 0.21 ± 0.8 which confirm the uniformity and homogeneity of LUVs in the mixture as well [32].

The value of zeta potential (ZP) proves the stability of the particulate systems. It is a measurement of the repulsive forces between the particles. Particles having a ZP of less than −30 mV or more than +30 mV are usually regarded as stable. Considering the ZP values were higher than −30 mV (Table 2), which confirms the acceptable stability of LUVs as well as their uniformity and size homogeneity suspension [18–20].

### 4.3. Construction of Calibration Curve

The following equation from the HPLC results was obtained:* Y* = 21998* X* + 8938, where* Y* is the area under the curve and* X* is the concentration of doxorubicin; the regression line of* R* ^2^ = 0.999 was obtained as well.

### 4.4. Entrapment Efficiency and* In Vitro *Drug Release

As seen from Table 1, in both formulations, liposomes contained maximum entrapment efficiency, nearly 100%, using the pH gradient technique [12, 13, 15].

**Figure 2:**
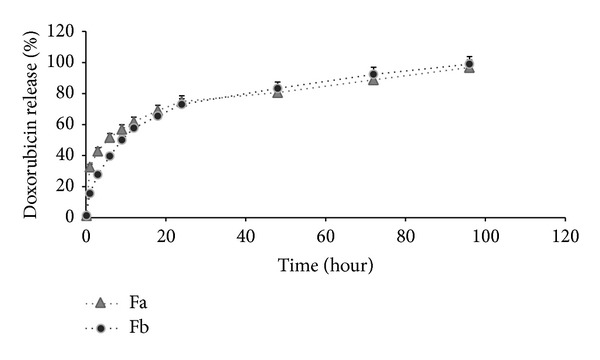
Cumulative release of doxorubicin in PBS (pH 7.4). Key: Fa (grey triangle), Fb (●).

Figure 2 shows the* in vitro* release of Dox during 96 hours where both formulations demonstrate a constant and continuous release profile. Since Fa and Fb liposomes include same ingredient with only difference in amount of PC and palm oil, they also have comparable releasing pattern with small variation. Within the first 6 hours, Fa demonstrated a faster release rate compared to Fb. During 6–24 hours, Fa and Fb liposomes showed almost similar release whereas, after 36 hours, Fa release goes slower than Fb; however no significant difference is observed.

### 4.5. Cellular Uptake and Cytotoxicity

**Figure 3:**
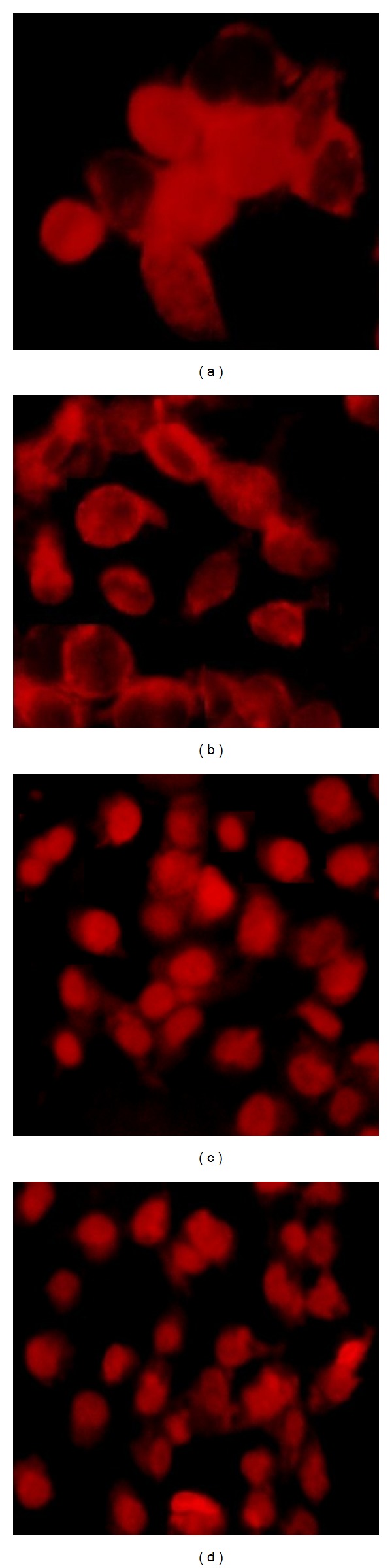
Cellular uptake of Dox liposome: (a) Fa liposome in MCF 7 cells, (b) Fb liposome in MCF 7 cells, (c) Fa liposome in MDA-MBA 231 cells, and (d) Fb liposome in MDA-MBA 231 cells.

Figure 3 demonstrates the cellular fluorescence images and cellular uptake of the Dox after the cells were incubated with liposome for 24 h. As Dox emits red fluorescence, the presence of Dox liposome can be clearly observed in MCF-7 and MDA-MBA 231 cells. After incubation of cell lines with Dox liposomes, they would cross the cell's membrane and the viable cells appear to have a red basis, while the apoptosis cells exhibited brighter reds, respectively [26–29].

As seen from Table 2, the IC50 of designed liposomal Dox and Caelyx^R^ on breast cancer cell lines were compared. In MCF-7 cells the IC50 for liposomal Dox with palm oil was 376.45 ± 9.20 and 387.22 ± 6.93 *μ*g/mL which is more effective than Caelyx^R^ with 483.84 ± 7.78 *μ*g/mL. Similarly, in MDA_MBA23 cells, the IC50 for designed liposome is 726.40 ± 7.58 and 755.73 ± 6.81 *μ*g/mL while it is 972.91 ± 9.87 *μ*g/mL for Caelyx^R^. In addition, potent IC50 in MCF-7 cell line indicates that MCF-7 cells are more sensitive to liposomal Dox than the MDA_MBA23 cells [25–29].

## 5. Conclusion

In order to take advantage of the therapeutic effects of palm oil, liposomal Dox formulations were prepared by replacing PC with different ratios of palm oil. Liposomal formulations containing 5% and 10% of palm oil were made through the freeze-thaw method, and then the TEM images revealed satisfactory morphology and formation of LUVs, respectively. Liposomal size distribution, zeta potential, and stability remain in the acceptable range. The HPLC results confirm the optimal drug loading through pH gradient technique and sophisticated* in vitro* release profile as well.

The finest cellular uptake was observed on MCF-7 and MDA-MBA 231 cell lines through 24 h; furthermore, cytotoxicity assay confirms the more effectiveness of the liposomal Dox containing palm oil in comparison to Caelyx^R^.

## Figures and Tables

**Table 1:** Particle size, zeta potential, and entrapment efficiency of the liposomes.

Formulation	Mean particle size (nm, ±SD)	Mean zeta potential (mV, ±SD )	Mean polydispersity index (PDI)	Mean entrapment efficiency (%, ±SD)
Fa	438.74 ± 1.9	−31.1 ± 2.6	0.22 ± 1.3	99.98 ± 3.18
Fb	453.71 ± 1.1	−32.2 ± 4.1	0.21 ± 0.8	99.99 ± 5.22

**Table 2:** IC50 of Fa, Fb, and Caelyx^R^ after 48 hours of treatment.

Formulation	IC50 MCF7 (*μ*g/mL, *n* = 3)	IC50 MDA-MBA 231 (*μ*g/mL, *n* = 3)
Fa	376.45 ± 9.20	726.40 ± 7.58
Fb	387.22 ± 6.93	755.73 ± 6.81
Caelyx^R^	483.84 ± 7.78	972.91 ± 9.87
